# The impact of COVID-19 pandemic on treatment adherence of patients with breast cancer

**DOI:** 10.1007/s00520-025-09582-8

**Published:** 2025-06-07

**Authors:** Nurit Betzer Lavee, Micha Barchana, Tuvia Baevsky, Israel Yoles

**Affiliations:** 1 Department of Management, Faculty of Social Sciences, Bar Ilan University, Ramat Gan, Israel; 2 Central District, Clalit Health Services, 30 Herzl St., Herzliya, Israel

**Keywords:** Breast cancer, COVID-19, Medication adherence, Patient compliance

## Abstract

**Purpose:**

Oncology patients may require long-term treatment adherence and may face additional challenges to adherence during national crises. This study investigated changes in treatment adherence among breast cancer patients before and during the COVID-19 pandemic.

**Methods:**

A historical prospective study was conducted using electronic medical records of adult breast cancer patients in the post-acute treatment phase; those receiving preventive oral monotherapy were analyzed. Adherence was assessed using the proportion of days covered (PDC).

**Results:**

The pre-pandemic baseline PDC among 4274 patients was 78.5%. During the four COVID-19 waves (Feb 2020–Oct 2021), patients with low pre-pandemic adherence (PDC < 75%) showed a significant increase in adherence (from 59 to 77%), while those with high pre-pandemic adherence (PDC < 75%) experienced a decline (from 94 to 88%). A statistically significant decline was observed from wave 1 (85.69%) to wave 4 (80.69%), *p < *0.001  . Older age was associated with higher adherence (*p*.(0.001 > Patients vaccinated during the third wave exhibited a significant decline in adherence between the first and fourth waves (*p < *0.001).

**Conclusions:**

This study emphasizes the need to monitor and support medication adherence among patients with chronic oncology conditions during health crises. The COVID-19 pandemic revealed shifts in adherence patterns, highlighting the urgency of improving healthcare preparedness, enhancing access to services, and ensuring treatment continuity in future emergencies.

Data from developed countries indicate that only about 50% of patients with chronic illnesses fully comply with their doctor’s recommendations [1]. Despite evidence that non-adherence to medication in chronically ill patients can lead to unexpected health outcomes, physiological imbalances, worsening of symptoms, and potentially new symptoms [2], non-adherence may also cause overutilization of the healthcare system, a higher patient load, and a subsequent considerable financial burden on the health system [3]. Hence, there is a great importance in identifying factors of non-adherence and developing methods to reduce it at both the individual and organizational levels.

Among the factors identified in previous studies that influence patient adherence to taking medication is the perceived threat of the disease [4–6]. When patients believe that their illness poses a serious threat to their health or carries a risk of imminent mortality, they are more likely to adhere to their prescribed treatment regimens, believing that adherence will protect them against the disease and its complications [5]. Conversely, when patients perceive their illness as less serious, they tend to be less adherent to treatment, which can lead to poor health outcomes and increased healthcare costs. This diminished perception of threat reduces the motivation to follow through with treatments, often exacerbating the condition and leading to greater financial burdens on the healthcare system [6].

## Breast cancer incidence and treatment

Though survival rates of breast cancer are increasing in many Western populations, it still accounts for a large percentage of cancer deaths around the world and is still the leading cause of death for women in many developed countries [7]. In Israel, in 2020, 18.4% of all cancer deaths were from breast cancer among Jewish women [8]. Although relative survival has improved significantly over the past decades and is now considered quite favorable, patients still face a long-term risk of recurrence after diagnosis. As a result, continued endocrine therapy is recommended for 3 to 10 years following initial treatment, depending on the tumor type and the woman’s menopausal status [9]. As this treatment may extend over years, breast cancer may become a de facto chronic illness with a potentially higher perceived threat than other chronic illnesses. This medication adherence has been found to be essential to prevent recurrence [3], yet reported adherence to adjuvant hormonal therapy among breast cancer patients varies, ranging from 50 to 80%, often declining over the course of the prescribed 5 to 10 years of treatment [10–12].

### Measuring adherence

There are several ways to effectively measure medical adherence effectively including patient self-report, pill count, and electronic measurements [3]. Recent advancements in electronic health records enable real-time analysis of medication adherence, which is shown to be more reliable than patient self-report [13]. The proportion of days covered (PDC), a pill count method, is preferred for its clarity and practicality, dividing treatment supply days by follow-up days. Compared to the medication possession ratio (MPR), which measures the percentage of days with available medication, PDC provides a more precise adherence metric [14]. This method is widely endorsed and has been validated through numerous studies [13, 15].

### The added threat of COVID-19 infection

The COVID-19 pandemic introduced a serious threat of a potentially fatal disease in early 2020, increasing the risk of mortality for chronically ill patients who were already at high risk. This global health crisis led to massive disruptions in healthcare services, particularly affecting those with chronic conditions due to restricted access to routine care and the redirection of medical resources to handle the surge of COVID-19 cases [16]. Additionally, the various closures and restrictions imposed to manage the epidemic significantly reduced access to medical services, further weakening the medical safety net for managing chronic illnesses and potential complications.

### Health protocols during the pandemic

In general, healthcare coverage in Israel is mandatory according to the National Health Insurance Law and is available to all Israeli citizens, including coverage of chronic illness treatment and any preexisting condition. Care is provided by four not-for-profit health maintenance organizations (HMOs). All members of the different HMOs have similar health insurance plans and access to health services, including low medication copayments [17].

The first wave of the pandemic began when the emerging virus reached Israel, from February 2020 until May 2020. The second wave occurred between June and October 2020, the third wave from November 2020 to February 2021, and the fourth wave started in July 2021 and lasted until October 2021 [18]. During the first two waves, prior to the widespread distribution of a vaccine, the Israeli Ministry of Health protocols enacted strict quarantines, curfews, and several national lockdowns [19]. During that time, cancer patients faced significant challenges in accessing care and medications [7]. The COVID-19 outbreak severely disrupted healthcare utilization among breast cancer patients, with many facing delays and cancellations of necessary treatments and follow-up appointments. This disruption added an additional layer of risk for these patients, already vulnerable due to their underlying health conditions [7, 20].

Despite what is known about the importance of treatment adherence and the paradox of adherence decline over time, evidence on long-term adherence patterns, particularly beyond five years among women receiving adjuvant hormonal therapy, remains limited. Moreover, almost nothing is known about treatment adherence during a pandemic. The goal of the present study was to assess the impact of the pandemic on longer-term treatment adherence for female breast cancer, focusing on monotherapy adherence (anastrozole, exemestane, letrozole, and tamoxifen).

## Methods

### Study population

The study was designed as a historical prospective cohort study using electronic medical records (EMR) from Clalit Health Services (CHS) Central District, the largest healthcare provider in Israel. Included in the analysis were all records of women previously diagnosed with non-metastasized breast cancer (ICD-9, 174.0–174.9; ICD 10, C50, C50.019–C50.919) within the district, who were taking oral monotherapy (anastrozole, exemestane, letrozole, and tamoxifen). Data for each participant was retrospectively gathered from October 2006 for record prescription and dispensing records, including the period prior to the outbreak of COVID-19 (February 2020) until the end of the fourth wave (October 2021). Inclusion criteria were female patients over age 18 in the post-acute treatment phase, where treatment was limited to preventive oral medication and routine medical follow-up.

### Data source

The EMR system of CHS included data from multiple sources: records of primary care physicians, community specialty clinics, hospitals, laboratories, and pharmacies. A registry of chronic disease diagnoses was compiled from these data sources using diagnosis-specific algorithms based on International Classification of Diseases Ninth Revision (ICD-9) code reading, text reading, laboratory test results, and disease-specific drug usage. The study protocols were reviewed and approved by Clalit Health Services’ Ethics Review Board Committee (COM2-0118–21) to ensure compliance with ethical standards in research. As a retrospective, blinded study, individual patient consent was not required; ethical compliance was ensured through IRB approval. All patient data were anonymized in accordance with the guidelines set forth by the data extraction committee, ensuring confidentiality and adherence to ethical research practices.

### Sample size and data items

Data were excluded for missing or incomplete records, and the final analysis included 4274 records. Variables included date of diagnosis, initial monotherapy prescription date, number of pills supplied and dispensed, data on COVID-19 infections, receipt of the COVID-19 vaccination, and general demographic data. Adherence rate was assessed using the proportion of days covered (PDC), as recommended by the Pharmacy Quality Alliance (PQA), calculated by dividing the number of days with treatment supply by the number of follow-up days [14].

### Statistical analysis

The collected data were processed using SPSS software (Chicago, v. 29). Pearson correlations were conducted to explore the relationships between date of initiation of prescription, date of initial dispensation, ongoing receipt of prescription, recurring date of dispensing, and age of the patient; *t*-tests were conducted to explore relationships between the percentage of PDC among those who were diagnosed with COVID-19 and those who were vaccinated, during the different COVID-19 waves. Specifically, a paired-sample* t*-test was performed to determine if there were significant differences in the PDC rates between the different waves. A one-way repeated measure ANOVA was performed to identify differences in dispensing patterns across various groups during different waves of the pandemic and across several time intervals, with follow-up Bonferroni analysis performed to further explore these differences. Analysis was conducted for each year of treatment during the pre-COVID period and the pandemic waves, calculating average PDC for all patients during that treatment year. An additional sub-analysis focused on the number of adherent patients, with adherence defined as the mean PDC of the pre-COVID treatment years, representing a reasonable benchmark for average adherence [21].

After computing the sample average of the PDC rate before the pandemic, we compared it with adherence rates found in the literature, selecting an average of 75% to define the adherence baseline. We then stratified the data during the four COVID waves into high > 75% and low adherence < 75% and analyzed changes in adherence for those two groups. To determine the sociodemographic level of the participants, we used the cluster formula for the characterization and classification of geographical units by the socio-economic status (SES) of the Israel Central Bureau of Statistics. This is a combination measurement that synthesizes demographic composition, years of education and schooling, standard of living, employment, and pension-aged percentages [22].

## Results

**Fig. 1:**
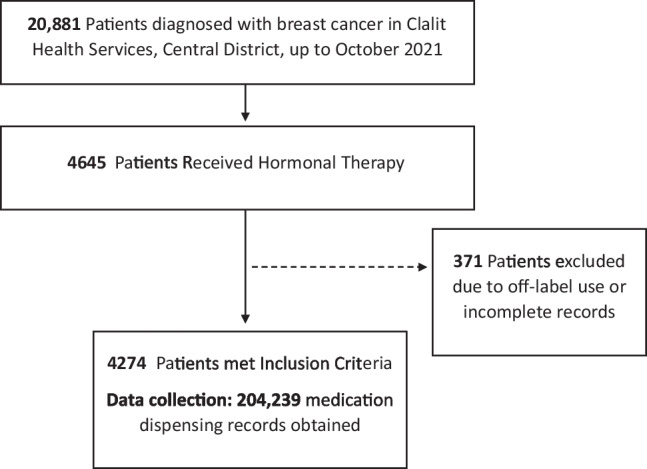
Participant flow diagram

There were complete prescription records for 4274 women participants (Fig. 1). Median age was 68.25 (29–98), where 17% were over 80 years of age. The most common medication prescribed was tamoxifen (61%) followed by letrozole (27%). The majority of the participants were classified at a medium SES, with 20% defined at a high SES, and 7% at the lowest socio-economic status (Table 1).

**Table 1:** Participant sociodemographic and behavioral characteristic**s**

Variable	Total (*N* = 4274)
**Age (years)**	
Median	68.25
Range	29–98
20–40	98 (2%)
40–60	950 (20%)
60–80	2824 (61%)
> 80	773 (17%)
**Prescribed hormonal therapy**	
Anastrozole	148 (3%)
Exemestane	31 (1%)
Letrozole	1260 (27%)
Tamoxifen	3206 (69%)
Socio-economic level*	
Low	338(7%)
Medium	3397 (73%)
High	910 (20%)
**Reported COVID-19 diagnosis**	
Yes	1667 (39%)
No	260 (61%)
**Received COVID-19 vaccination**	
Yes	3978 (93%)
No	299 (7%)

*As determined by the ICBS cluster analysis formula [22]

Thirty-nine percent of women included in the analysis were diagnosed with COVID-19 infection at some point during the four recorded waves, and 93% received the COVID-19 vaccine after it became available in December of 2020 [23] (Table 1).

## Adherence rates prior to the pandemic

**Fig. 2:**
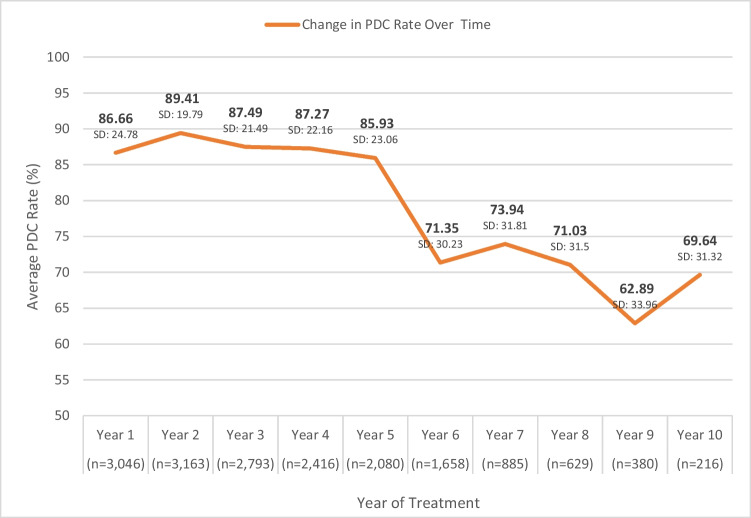
The proportion of days covered (PDC) average for each year of treatment prior to the four waves of the pandemic

To determine the average adherence rate prior to the pandemic, we stratified the data according to years in treatment, and then calculated the means of PDC for each year. The full sample had up to 10 years of treatment follow-up, with a PDC average of 79.3%. Prior to the COVID-19 epidemic, the average PDC rate declined over time in treatment from the second year, with a significant decline after year 5 (62.89%–89.41%) (Fig. 2). Age was also positively correlated with adherence, finding that the older the patient, the higher the adherence rate. However, this correlation was only statistically significant from year 1 to year 5 of treatment (*p* < 0.001). No significant differences in PDC rates were noted in relation to sociodemographic level.

### Adherence behavior during the pandemic

**Fig. 3:**
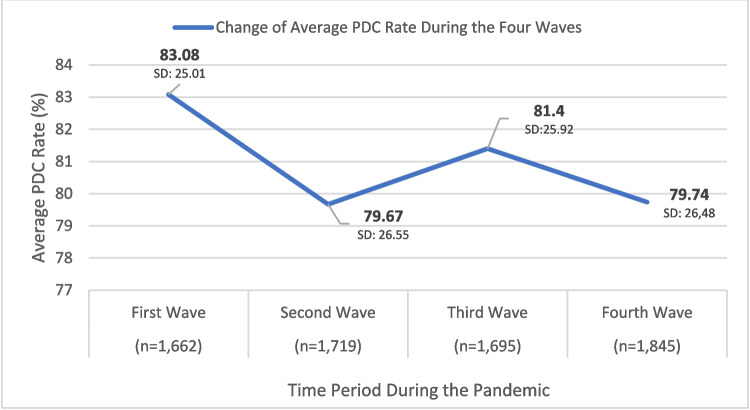
The PDC rates during the four COVID-19 waves

During the pandemic, overall adherence rates increased, with PDC rates ranging from 79.7% to 83% (Fig. 3). The same correlation between age and adherence was noted, as seen prior to the pandemic. During all four waves, the correlation was significant (*p* < 0.01 or *p* < 0.001).

To better compare adherence behavior, we divided the participants into two categories of either high or low adherence, where the cutoff point was 75%. The rate of 75% was chosen based on both the literature, which indicates that adherence rates typically range between 50 and 80% [10–12], and our own pre-pandemic overall average adherence rate of 79.3%.

**Fig. 4:**
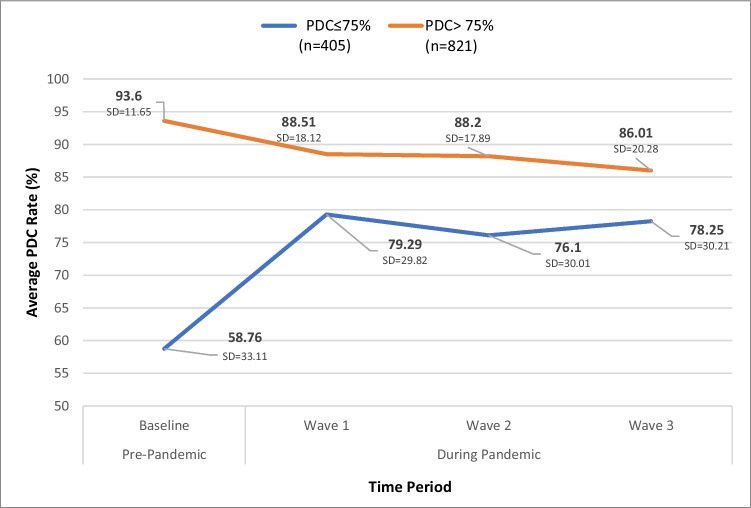
Comparison between PDC rates prior to COVID-19 epidemic with subsequent COVID-19 waves. Patients with below 75% (PDC ≤ 75%) and above 75% (PDC > 75%)

Of the 405 participants who had less than 75% PDC rate prior to the COVID-19 outbreak, there was a significant increase in PDC rates from 58.76% before the pandemic to 79.2% during the first wave and maintained similar rates in subsequent waves (*F*(3,1212) = 76.28, *η*^2^ = 0.159, *p* < 0.001) (Fig. 4). Conversely, the 821 participants who had a high PDC rate prior to the pandemic (> 75%) showed a significant decrease from a pre-pandemic rate of 93.60% to the first wave (88.51%), with further slight decreases in subsequent waves (*F*(3,2460) = 31.51, *η*^2^ = 0.037, *p* < 0.001). While both low and high adherence groups showed significant changes, the group with a PDC rate < 75% demonstrated a more substantial improvement (20.53% increase) when compared to the participants who had over 75% PDC group’s decline (5.09% decrease). These changes persisted throughout all waves of the pandemic**.**

### Correlation between adherence behavior, COVID-19 vaccination, and COVID-19 diagnosis

To compare medication adherence behavior during the pandemic, we examined PDC rates of patients who had been diagnosed with COVID-19 and those who had received their first vaccine dose. A significant difference was found when comparing the first and fourth waves for individuals vaccinated during the third wave. A paired *t*-test revealed a statistically significant decrease in PDC rates (*t*(1219) = 6.16, *p* < 0.001). The mean PDC rate in the first wave (*M* = 85.69%, SD = 23.17) was significantly higher than in the fourth wave (*M* = 80.69%, SD = 26.05). For patients diagnosed with COVID-19 during the second or third waves, no significant changes in PDC rates were observed between pre- and post-diagnosis waves. Similarly, no significant differences were found in PDC rates between the second and fourth waves for the vaccinated group.

## Discussion

Our study on breast cancer patient adherence to oral monotherapy during the COVID-19 pandemic in Israel reveals several important findings that both align with and diverge from previous research on medication adherence of high-threat chronic illness. A study conducted on a similar geographic population in Israel, of breast cancer patients prior to the COVID-19 pandemic, using a hospital population, found an overall PDC rate of 82%, with similar correlations between younger age and reduced adherence [24]. Our findings of an overall average PDC of 79.3% before the pandemic are comparable, suggesting consistency in adherence patterns among breast cancer patients in Israel. Just as we found in our results, the study in Israel [24], as well as other larger analyses in other countries, has found that adherence also declines with years from initial treatment [25]. It is important to note that the average recommended time for adjuvant hormonal therapy in breast cancer treatment is 5–10 years, depending on the specific case and type of medication [26]. This extended treatment duration may make maintaining adherence particularly challenging, as evidenced by the decline in adherence rates over time observed in our study and others.

We did see an initial significant increase in treatment adherence during the first wave of the COVID-19 pandemic. This finding aligns with studies from other countries that reported increased medication adherence among chronically ill patients during the early stages of the pandemic. A study in the USA found improved adherence to cardiovascular medications during the initial months of the pandemic [27]. Similarly, research in China reported increased adherence to antihypertensive medications during the early stages of the COVID-19 outbreak [28]. We cannot know from our data alone why this increase in adherence occurred. However, the concept of “systemic shock” may be offered to explain these behavioral changes. The COVID-19 pandemic represented a sudden, large-scale disruption to normal life and healthcare systems [29], potentially serving as a wake-up call for patients regarding the importance of their health and medication adherence. This systemic shock may have heightened patients’ perception of health threats, leading to improved adherence behaviors, particularly among those who were previously less adherent. Our findings are particularly interesting when comparing to a pre-pandemic study on adherence to hormonal therapy among Israeli breast cancer patients [24], which found that adherence rates in Israel were generally high, with about 84% of patients considered adherent (PDC ≥ 80%). However, they also noted that younger age was associated with lower adherence, which aligns with our findings both before and during the pandemic.

The COVID-19 pandemic seems to have had multifaceted effects on breast cancer patient behavior. While we observed an initial increase in adherence, particularly among previously non-adherent patients, the impact was not uniform across all groups. The decrease in adherence among previously adherent patients, although smaller in magnitude, suggests that the pandemic’s effects on healthcare behaviors were complex and varied. Based on previous studies reviewing behavioral influences on adherence patterns, several factors may have been involved. As researchers have suggested, the pandemic may have increased a sense of overall fear, including fear for the body and fear of not knowing, domains that are activated by a perceived threat [30], which could have potentially motivated previously non-adherent patients to improve their medication-taking behavior [31]. While fear may have increased adherence for some, healthcare disruptions during the pandemic may have made it more challenging for others to maintain their usual level of adherence, depending on whether their fear of contracting COVID-19 was a greater threat than coping with the cancer diagnosis [3]. A systematic review found that, in seven studies, adherence to therapy declined during the pandemic period compared to a control period in prior years, while five other studies reported either no change or an improvement in adherence levels during this period [32], demonstrating that many factors are at play in influencing adherence. Finally, stress and anxiety associated with the pandemic may have affected patients differently, potentially improving adherence in some while having a negative impact on others [33].

### Clinical and research implications

The observed changes in adherence patterns following vaccination, COVID-19 diagnosis, age, treatment duration, and variations across different waves may reflect broader treatment-related responses among patients receiving long-term therapy for chronic illness. Notably, a decline in adherence rates was observed, including among vaccinated individuals between the first and fourth waves. While this trend could potentially be related to a reduced sense of urgency or perceived threat following vaccination, it is important to note that our study did not directly measure perceived threat. Other factors such as reduced healthcare access, pandemic fatigue, or broader systemic disruptions may also have contributed to this change. Additionally, older patients consistently demonstrated higher adherence levels, although a decline was observed after five years of treatment. These findings highlight the potential role of age and treatment duration in shaping long-term adherence.

### Limitations

Almost 400 patients were removed from the analysis because they were prescribed dosing of the medications that varied from recommended protocols or had incomplete data records. Additionally, we were not able to retrieve data prior to 2006. The large sample was taken from the center of the country, which overall had only a small percentage of participants from lower or higher socioeconomic levels, so comparison among these groups was limited. Nor was specific sociodemographic information such as ethnicity or religion available. Further research should include a wider variety of participants, including ethnicity and economic status, as well as geographical location within the country. Initially, patient questionnaires were created to complement data and better understand reasons for compliance/noncompliance. However, by the time the necessary approvals for the study were obtained, it was no longer feasible to distribute these questionnaires at the appropriate time. This limitation affected our ability to capture patients’ immediate perceptions and behaviors, which could have provided valuable insights into factors influencing adherence during key phases of the study.

Non-adherence to medication may be influenced by a wide range of factors, including, but not limited to, comorbid conditions, adverse effects, socioeconomic status, and psychological distress. Given the scope and design of this study, it was not feasible to isolate and statistically adjust for each of these variables individually. Instead, we focused on within-subject behavioral trends by comparing each patient’s adherence patterns before and during the pandemic. This approach enabled us to assess whether a sudden and substantial change in medication-taking behavior occurred at the onset of the COVID-19 pandemic —a well-defined and temporally distinct event. We believe that observing such rapid shifts in adherence within the same individuals offers reasonable grounds to attribute the behavioral change, at least in part, to the pandemic context.

## Conclusions

This study demonstrates that the COVID-19 pandemic had a significant, yet nuanced, impact on medication adherence among breast cancer patients in Israel. The findings highlight the complex interplay between external health threats and long-term health behaviors. As we continue to navigate the post-pandemic landscape, these insights can inform strategies to maintain and improve medication adherence among chronically ill patients, particularly in times of health crises.

Finally, there are several suggestions for future studies to investigate how treatment adherence can be supported during future public health and national crises. (1) Future research should investigate post-pandemic adherence behaviors to assess whether lasting changes in adherence patterns are evident. For example, a recent study on tuberculosis patients found that reduced adherence rates remained at a lower rate, post pandemic [34]. It is important to understand whether this is disease and country dependent or could be part of a larger trend which must be addressed by future healthcare policy. (2) Understanding which factors are perceived as threats to patients’ health could be leveraged to improve adherence among those diagnosed with life-threatening illnesses. However, future research is needed to explore whether certain sociodemographic factors influence adherence in crisis situations and how best to target these groups. (3) Additional studies are needed to emphasize how adherence could be strengthened during national crises, like a pandemic or war. For example, the ongoing war in Ukraine has necessitated new approaches to medication delivery, creating a conceptual model for providing reliable pharmaceutical care while reducing the burden on the healthcare system [35]. The results of the current study contribute to increased understanding of patient adherence during times of intense crisis, to better implement these types of changes within health system policy, enabling improved overall preparation for national and international healthcare crises. Together, these research directions could help build a more resilient healthcare infrastructure that supports adherence during crises and fosters long-term improvements in patient outcomes across diverse populations and settings.

## Data Availability

No datasets were generated or analysed during the current study.
