# Correction to: The impact of COVID‑19 pandemic on treatment adherence of patients with breast cancer

**DOI:** 10.1007/s00520-025-09724-y

**Published:** 2025-07-10

**Authors:** Nurit Betzer Lavee, Micha Barchana, Tuvia Baevsky, Israel Yoles

**Affiliations:** 1 Faculty of Public Health Management, Bar Ilan University, Ramat Gan, Israel; 2 Central District, Clalit Health Services, 30 Herzl St., Herzliya, Israel

**Correction to: Supportive Care in Cancer (2025)**

10.1007/s00520-025-09582-8

In the last cell of the Table 1 (row 19, column 2), the value appears as 206 instead of the correct number 2067.

**Table 1:** Participant sociodemographic and behavioral characteristic**s**

Variable	Total (N = 4,274)
**Age (Years)**	
Median	68.25
Range	29–98
20–40	98 (2%)
40–60	950 (20%)
60–80	2,824 (61%)
> 80	773 (17%)
**Prescribed hormonal therapy**	
Anastrozole	148 (3%)
Exemestane	31 (1%)
Letrozole	1,260 (27%)
Tamoxifen	3,206 (69%)
Socio-economic Level*	
Low	338(7%)
Medium	3,397 (73%)
High	910 (20%)
**Reported COVID-19 Diagnosis**	
Yes	1667 (39%)
No	2607(61%)
**Received COVID-19 Vaccination**	
Yes	3, 978(93%)
No	299 (7%)

*As determined by the ICBS cluster analysis formula [22]

The correct Table 1 is shown below.

The original article has been corrected.

## Data Availability

The data that support the findings of this study are not publicly available due to privacy restrictions.

